# HIV Suppression among Patients on Treatment in Vietnam: A Review of HIV Viral Load Testing in a Public Urban Clinic in Ho Chi Minh City

**DOI:** 10.1155/2011/230953

**Published:** 2011-02-07

**Authors:** T. Tony Trinh, Brian T. Montague, Timothy P. Flanigan, Hoang My Gerard

**Affiliations:** ^1^Department of General Internal Medicine, Alpert School of Medicine of Brown University, 593 Eddy St., Providence, RI 02909, USA; ^2^Division of Infectious Diseases, Alpert School of Medicine of Brown University, 164 Summit Avenue Rise Building 148, Providence, RI 02906, USA; ^3^Médicins du Monde, Ho Chi Minh City, Vietnam; ^4^Management Sciences for Health/Supply Chain Management System (MSH/SCMS), 25 Bui Thi Xuan, Hanoi, Vietnam

## Abstract

*Background*. There are few reports of HIV viral load (VL) testing among patients on ART in Vietnam. *Methods*. From a public clinic in Ho Chi Minh City (HCMC), we reviewed cases of VL measurements from adults on ART. *Results*. We identified 228 cases. Median age was 30 years (27–34), 85% were male, and 77% had a history of IDU. The mean ART duration was 26 months (95% CI 25–27); d4T/3TC/NVP was the most common regimen. Viral suppression was seen in 160/228 (70%). Viremia (>1000 copies/mL) was associated with prior ART exposure (OR 5.68, *P* < .0001) and immunologic failure (OR 4.69, *P* = .0001). Targeted testing accounted for 13% of cases, only half of which yielded viremia. *Conclusion*. We demonstrate a high HIV suppression rate among patients on ART in HCMC, Vietnam. In this setting, routine testing detects viremia missed by targeted testing.

## 1. Introduction

Vietnam is a country with one of the highest prevalences of HIV in Southeast Asia. With an estimated prevalence of 293 000 people in 2007 (approximately 0.5% of the general population), the HIV epidemic is primarily concentrated in urban areas among key high-risk populations, the majority of which are injection drug users (IDU), and to a lesser extent, female sex workers (FSWs) and men who have sex with men (MSM) [1, 2]. Efforts to confront the HIV epidemic in Vietnam face a high burden of patients with comorbid substance abuse and limited resources. 

International efforts to scale up antiretroviral therapy (ART) have greatly improved funding for treatment in Vietnam, allowing for approximately 14 969 people to receive ART as of 2007 [3]. The success of ART programs has been documented in resource-limited settings throughout the world [4]. However, cohorts examining the effectiveness of ART programs in low-income countries traditionally consist of countries with a low prevalence of IDU, the majority of which are in Africa [5, 6]. Thus, there is less information regarding ART scale-up efforts in resource-limited settings with a high burden of comorbid IDU. Only since 2009 has the first report of ART among IDU in Vietnam been documented [7]. 

Virologic suppression is the measure of successful antiretroviral therapy. The cost of viral load monitoring, however, has been prohibitive in resource-limited settings.. The World Health Organization (WHO) has recommended an algorithm using clinical and immunologic criteria to assess treatment failure in the absence of viral load testing which has become standard of care in many resource-limited settings [8]. The concern has been raised that delayed recognition of treatment failure may lead to prolonged use of failing regimens and amplification of drug resistance [9]. This concern may be most important in populations at highest risk for failure including those with active substance abuse. 

At the An Hoa Outpatient Center (OPC), a public urban HIV clinic located in District 6 (D.6) of Ho Chi Minh City (HCMC), Vietnam, viral load (VL) testing has been available in a limited capacity since 2005. Virologic testing has been used principally as a confirmatory test targeted at patients suspected of failing based on WHO criteria of clinical or immunologic failure. In December 2007, as part of a quality improvement process, a program of routine viral load surveillance was implemented at the An Hoa OPC. The goals of this study were to document ART efforts in a resource-limited setting with high prevalence of IDUs, to identify high-risk groups for failure who may benefit from more frequent monitoring or other interventions and to assess the potential for delayed diagnosis of treatment failure when using targeted testing based on clinical and immunologic criteria. 

## 2. Methods

### 2.1. Site and Population

Located in District 6 (D.6), An Hoa Outpatient Center (OPC) is funded by USAID and the US President's Emergency Plan for AIDS Relief (PEPFAR) and has been operated by the French nongovernmental organization Médicins du Monde (MdM) since 2003. An Hoa Outpatient OPC is one of 75 PEPFAR clinical sites, with additional oversight by the Vietnamese Ministry of Health (MOH), that provides ART- and HIV-related services to one of the HCMC's 18 inner city districts. 

Available first-line three-drug ART regimens are consistent with WHO standards which include two nucleoside reverse transcriptase inhibitors (either stavudine (d4T) + lamivudine (3TC) or zidovudine (AZT) + lamivudine (3TC)) with one non-nucleoside reverse transcriptase inhibitor (either nevirapine (NVP) or efavirenz (EFV)) [8]. All patients preparing to initiate ART through the An Hoa OPC must undergo pretherapy adherence counseling sessions. Once initiated, patients are scheduled to undergo clinical assessments with a physician and nursing at weeks 2, 4, 8, 12, 16, 24, 32, 40, and 48. Routine visits are scheduled every 2 to 6 months afterwards. Per MOH guidelines, once stable on ART, patients must come to the OPC monthly to receive their ART supply. Additionally, patients are seen by an adherence counselor at weeks 2, 4, 8, 24, and 48. CD4 counts are routinely obtained 12, 24, and 48 weeks and every 6 months afterwards. 

Treatment failure is suspected if after one year of therapy, patients exhibit immunologic failure (fall to baseline level CD4 count, 50% fall in CD4 count from posttreatment peak, or CD4 counts persistently <100/mm^3^) and/or clinical failure (new or recurrent WHO stage IV condition) according to WHO criteria [10]. Switching to second-line regimen involves a thorough committee review by specialists at the Hospital for Tropical Disease in Ho Chi Minh City. 

Viral load (VL) testing is available at the An Hoa OPC in a limited capacity. During the dates from 12/1/2007 to 2/28/2009 VL testing was performed according to two distinct approaches the following.

1. Targeted testing to confirm virologic failure among those suspected of treatment failure and who were in consideration for 2nd-line therapy. This approach was done with funding from the US CDC based on the Vietnamese Ministry of Health and is available to all PEPFAR sites in HCMC.
2. Routine testing to screen for subclinical virologic failure for those on ART for greater than one year. This approach was internally funded through MdM and has been in practice since 12/1/07. Under this approach, patients established on ART for greater than one year received one-time VL testing as part of a routine visit (as stated above).

All VL samples were sent to an offsite facility at the Pasteur Institute of HCMC, where they were assessed based on real-time reverse transcriptase PCR assay (Generic HIV viral load assay, Biocentric, Bandol, France) with a threshold for detection of VL of 250 copies/mL. 

### 2.2. Data Collection

We reviewed cases of VL testing performed at the An Hoa OPC between the dates of 12/1/07 and 2/28/09. Included for review were adult patients (>18 years) on ART for greater than one year while actively registered at the An Hoa OPC. We excluded patients younger than 18 years of age, and those who had been on ART for less than one year in duration from date of registration to An Hoa OPC. 

An onsite database managed by trained nursing staff was used to identify cases for review. In situations where multiple VL measurements were sequentially performed on a single patient, the initial date of VL testing was used for case identification. 

Medical records were reviewed and abstracted for demographic data (e.g., age and sex), self-reported history of IDU, date of registration to the AnHoa OPC, date of ART initiation, 1st-line ART regimen, duration of ART at time of VL testing, baseline CD4, CD4 at time of VL testing, prior ART exposure, adherence, history of immunologic and clinical failure, switch to 2nd-line regimen, and approach used to perform VL testing, that is, targeted or routine (nontargeted). Current and prior history of IDU was not distinguished. 

Significant viremia was designated as a VL of greater than 1000 copies per milliliter. Virologic failure, as defined by the WHO, was designated as VL greater than 10 000 copies/mL. 

Prior ART exposure was defined as prior ART usage not under the supervision of any MOH monitored site. Immunologic failure was determined according to WHO standards, that is, fall to baseline level CD4, 50% fall posttreatment peak, or levels persistently <100/mm^3^ after one year of ART. Clinical failure was determined by new or recurrent WHO stage IV condition. Adherence was designated as “Good” if patient had >95% self-reported adherence rate in the preceding month, and “Poor” if the patient had <95%. 

Descriptive statistics were generated for demographic and clinical parameters. Univariate associations between demographic and clinical factors and the presence of significant viremia were tested by means of chi-square statistics. Multivariate logistic regression was used to control for potential confounding factors. 

This paper was approved by the Provincial AIDS Committee (PAC), Ministry of Health (MOH) of Ho Chi Minh City, Vietnam. 

## 3. Results

**Figure 1:**
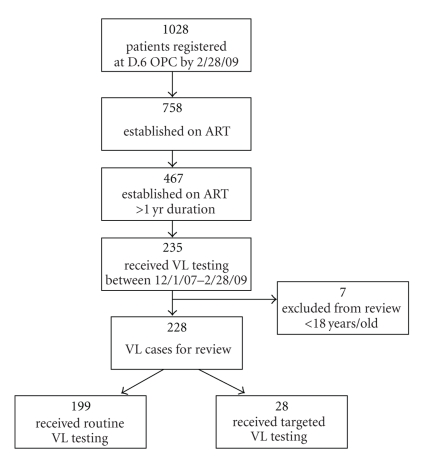
Flow chart of adult patients on ART >1 yr who received VL testing and enrolled for review.

As of the end of February 2009, a total of 1028 patients were registered as active at the An Hoa OPC. Seven hundred and fifty-eight patients were receiving ART, and 467 of whom had been on ART for greater than one year's duration. We identified 235 unique cases of viral load measurements for review. Seven were excluded because of age less than 18 years yielding a total of 228 for our study (Figure 1). 

Composite baseline characteristics are outlined in Table 1. The majority of patients were male (85%) and had a history of IDU (77%). The median age was 30 years (interquartile range 27–34). The regimen of d4T/3TC/NVP was the most common 1st-line regimen utilized (65%), and 74% had documented Good adherence. Thirty percent had prior ART exposure not under the supervision of a MOH monitored site. The median CD4 count at time of registration to the An Hoa OPC and time of VL testing was 57 cell/*μ*L and 239 cells/*μ*L, respectively. The mean duration of ART prior to VL testing was 26 months (95% CI 25–27), and undetectable virus was exhibited in 160 of 228 cases (70%). Measurements below our designated threshold of significant viremia (1000 copies/mL) were seen in 175 of 228 cases (77%).

In univariate analysis the odds of significant viremia were higher for those with prior ART exposure (*P* < .0001), preceding immunological failure (*P* < .0001), clinical failure (*P* = .024), and female sex (*P* = .0428) (Table 2). In multivariate analysis, two factors remained strongly associated with significant viremia: prior ART exposure (OR 5.69, *P* < .001) and history of immunologic failure (OR 4.69, *P* = .0001). There was an observed trend towards increased viremia among women, but not enough women were enrolled to find a significant difference (*P* = .066).

A comparison of targeted and routine testing is seen in Table 3. Of all cases yielded for review, 13% (29) came as a result of targeted testing. Only half (48%) of cases targeted for testing yielded significant viremia (>1000 copies/mL), and all but one of which had virologic failure (>10,000 copies/mL). The sensitivity of targeted testing in detecting significant viremia was 26% with a positive predictive value of 50%. Approximately 80% of those targeted had immunologic failure, 72% had prior ART exposure, and 21% had clinical failure, compared to 7%, 11%, and 4% of those who had routine testing, respectively. 

## 4. Discussion

In our study, viral load testing was performed on 49.7% of adult patients on ART for greater than one year attending the An Hoa OPC, and 77% of whom had a history of IDU. The mean duration of ART was 26 months, and the rate of undetectable virus and viremia below 1000 copies/mL was 70% and 77%, respectively. 

The detectable threshold of our assay, 250 copies/mL, is one that is not commonly used in studies evaluating ART efficacy. A detectable threshold of 400 copies/mL is what has been traditionally used in assays from studies conducted in resource-limited settings. In studies from Africa, the viral load suppression rate has ranged between 66% and 82% for patients on ART for duration of 26–48 weeks [11–13]. Our study found a comparable suppression rate at a longer mean ART duration, 26 months, despite using a lower level of detection (i.e., more sensitive assay), in a predominantly IDU population. Since our study excluded patients on ART for less than one year, there may have been a selection bias towards patients more tolerant of and more compliant with ART which could partially explain the relatively high viral suppression rate observed.

Injection drug users have often been associated with lower rates of virologic suppression [14, 15]. This is traditionally attributed to incomplete adherence, and the psychosocial instability that comes with drug-seeking behavior [16, 17]. International treatment cohorts which have documented the efforts of ART programs in low income countries (ART-LINC) have excluded resource-limited countries with higher rates of IDU [5, 6]. Amongst developing nations, China and Russia have been estimated to have the highest rates of IDU [18]. Recently, the first study examining viral load suppression rates among 8 ART programs in China revealed a VL suppression rate of 67% for patients on 24 months of ART [19]. However, IDUs may have been underrepresented in this study, as IDU associated transmission was reported in only 8% of patients. Studies documenting the VL response of ART programs in Russia and Eastern Europe remain to be seen. 

The program at the An Hoa OPC demonstrates a successful campaign of viral suppression among an HIV population with a high prevalence of IDU. The viral suppression rate in the current study is similar to that seen in a cohort of HIV positive drug users on ART in Hanoi, Vietnam [7]. It is notable however that IDU history was not statistically associated with viremia in our study. This observation perhaps is due to the lack of distinction between current and former IDU activity in our patients. Former IDUs have been shown to have similar VL suppression rates to non-IDU patients on ART [14].

The observed trend towards increased viremia among women is somewhat surprising in our study. It has been noted that the HIV epidemic among women in Vietnam has been greatly under-reported and under-recognized. Institutional efforts focus primarily on young male injection drug users, leaving women not only less likely to get tested, but also less likely to receive optimal care [20]. A combination of several cultural factors, including stigma directed against HIV-infected patients, poor education, and reluctance to seek medical care, subordinate gender roles, may also work to create a significant barrier to the optimization of medical care for HIV-infected women in Vietnam [21–23]. However, given the relatively low number of women in our cohort, our findings need to be interpreted with caution. Further studies are needed to adequately address variables such as gender differences in patterns of clinical utilization that may be contributing to failure among HIV-infected Vietnamese women on ART. 

Prior unmonitored ART exposure was shown to be a significant risk factor for treatment failure in our study. Patients in developing countries with a history of unmonitored ART usage are at risk of improper administration of medications as well as exposure to substandard or counterfeit drugs. These patients are at risk not only for harmful side effects but also for the development of HIV drug resistance. Our data adds to the previous studies which suggest that patients with prior ART should be more closely targeted for suspected ART failure [24]. 

The significant association of immunologic failure and viremia in our study is not surprising. The history of HIV shows that immunologic failure naturally follows progressive sustained viremia. Virologic failure predates immunologic failure, which is followed by clinical failure. Thus, strictly using immunologic and clinical monitoring, that is, the WHO algorithm, as a method to identify ART failure will invariably miss early virologic failure thus allowing for extended viral replication under drug pressure and promote drug resistance. 

Multiple studies have shown that the application of the WHO algorithm is a poor substitute for viral load testing, lacking sensitivity and specificity for detecting treatment failure in resource-limited settings [12, 24–29]. Furthermore, in settings with no capacity for viral load testing, strictly using the WHO algorithm also leads to potential misclassification of treatment failure and premature switch to second-line regimens [26, 30]. A recent study evaluating immunologic monitoring in a resource-limited setting, found that only 42% of patients qualifying for immunologic failure had detectable virus [26]. Our study also found a potential for misclassification as only 48% of those targeted had significant viremia. 

The goal of targeted VL testing, as adopted by the Vietnamese MOH in HCMC, is to reduce the likelihood of premature switch to valuable second-line regimens. However, as our study shows, given the insensitivity of the criteria used for targeted testing, treatment failure may be under-diagnosed and opportunities to intervene early will be lost. 

Our review suggests that routine testing has the potential to identify patients with significant viremia not identified by targeted testing programs and thus prevent delayed recognition of treatment failure. Implementing routine testing, however, poses a number of logistical barriers [31]. Decisions regarding the use of virologic monitoring need to consider the cost, frequency, and availability of follow-up testing along with the risk of reducing access to treatment or other necessary health services. Other factors that need to be considered are the threshold for change in regimen and the role of adherence interventions. 

A major limitation in our study was the usage of single viral load measurements. Low level viremia in our review is difficult to distinguish from “blips” clinically insignificant episodes of nonsustained, transient low level viremia [32]. We designated a level of 1000 copies/mL to represent significant viremia, as levels above this threshold have been shown to be frequently associated with resistance, often leading to therapy changes [31, 33]. However, using this threshold, we potentially excluded those who may have been experiencing persistently low viremia, thus possibly under-diagnosing treatment failure. 

Additionally, single viral load measurements are challenging to interpret without the appropriate infrastructure for followup, that is, subsequent testing, resistance analysis, and resources to target adherence. Our study was not designed to assess followup, but data from our review may be used in designing a protocol for follow-up testing and targeted interventions. Targeted adherence interventions have been shown to be successful in reducing viral load breakthrough to undetectable levels in a resource-limited setting [34]. 

## 5. Conclusions

In summary, we demonstrate a successful campaign of HIV viral load suppression in HCMC, Vietnam, a resource-limited area with a high prevalence of IDUs. We found that An Hoa OPC patients well established on ART experienced a high viral load suppression rate comparable to that seen in other studies. Significant viremia was strongly associated with immunologic failure and prior ART exposure. A trend towards increased viremia was observed among women on ART that warrants further investigation. Targeted testing, based on the WHO algorithm, was a poor predictor of virologic failure. Routine screening is better able to identify patients on ART who experience significant viremia. This approach however requires a comprehensive structure for followup and intervention; the costs of which need to be considered on a site by site basis in resource-limited areas. 

## Figures and Tables

**Table 1:** Baseline characteristics of patients on ART >1 yr who received VL testing, *N* = 228.

Characteristic	
Male (%)	193 (85)
Female (%)	35 (15)
Age at study, median (interquartile range), years	30 (27–34)
Age at ART initiation, median (interquartile range) years	27 (24–31)
IDU (%)	175 (77)
Prior ART exposure (%)	30 (13)
Duration of ARV at time of viral load testing, mean (95% CI), months	26 (25–27)
Adherence: “Good” (%)	169 (74)
Adherence: “Poor” (%)	59 (26)
First-line regimen	
AZT/3TC/EFV	4 (2)
AZT/3TC/NVP	14 (6)
D4T/3TC/EFV	61 (27)
D4T/3TC/NVP	149 (65)
CD4 cell count, median (interquartile range) cells/*μ*L	
at registration to An Hoa OPC	57 (18–146)
at viral load testing	240 (144–366)
Documented immunologic failure (%)	39 (17)
Documented clinical failure (%)	14 (6)
Switch to 2nd line* (%)	13 (6)
VL testing	
according to targeted approach (%)	29 (13)
according to routine approach (%)	199 (87)

IDU: injection drug use, VL: viral load.

ART: antiretroviral therapy.

AZT: zidovudine, D4T: stavudine, EFV: efavirenz.

NVP: nevirapine, 3TC: lamivudine.

*Second-line regimen: tenofovir (TDF)/lamivudine (3TC)/lopinavir (LPV).

**Table 2:** Characteristics of patients on ART >1 year according to result of VL testing, threshold VL 1000 copies/mL.

Characteristics	VL < 1000 (*N* = 175)	VL > 1000 (*N* = 53)	OR (95% CI)	*P* value	
				Univariate	Multivariate
Gender: male (%)	153 (87)	40 (75)	0.44 (0.2–0.95)	—	—
Gender: female (%)	22 (13)	13 (25)	2.26 (1.05–4.88)	.043	.066
IDU (%)	133 (175)	42 (79)	1.2 (0.57–2.55)	.34	—
Prior ART exposure (%)	12 (7)	18 (34)	6.99 (3.09–15.8)	<.0001	<.0001
Adherence: “Good” (%)	132 (75)	37 (70)	0.75 (0.38–1.49)	.419	—
Adherence: “Poor” (%)	43 (25)	16 (30)	1.33 (0.67–2.62)	—	—
CD4 cell count, median (interquartile range) cells/uL					
at time of registration to An Hoa OPC	59 (20–143)	50 (12–149)	—	—	—
at time of viral load testing	267 (171–374)	167 (78–260)	—	—	—
Documented immunologic failure (%)	19 (11)	18 (34)	4.2 (2.0–8.9)	<.0001	.0001
Documented clinical failure (%)	7 (4)	7 (13)	3.6 (1.2–10.9)	.024	—

IDU: injection drug use, ART: antiretroviral therapy.

OPC: outpatient center, VL: VIRAL load.

**Table 3:** Characteristics of patients on ART >1 yr—targeted testing and routine testing.

Characteristic	Targeted testing (*N* = 29)	Routine testing (*N* = 199)
CD4 cell count, median (interquartile range) cells/*μ*L		
at time of registration to An Hoa OPC	45 (14–107)	59 (18–149)
at time of VL testing	78 (53–93)	267 (179–400)
Documented immunologic failure (%)	24 (83)	14 (7)
Documented clinical failure (%)	6 (21)	8 (4)
Switch to 2nd line (%)	9 (31)	3 (2)
Undetectable or VL < 1000 copies/mL (%)	15 (52)	160 (80)
Significant viremia (copies/mL)		
High viremia: VL > 10000 (%)	13 (45)	14 (7)
Moderate viremia: 10000 > VL > 1000 (%)	1 (3)	25 (13)

ART: antiretroviral therapy.

OPC: outpatient center, VL: viral load.
